# Methods to Increase or Decrease Resistance to Photodegradation and Biodegradation of Polyurethane/Polyisocyanurate (PU/PIR) Foams

**DOI:** 10.3390/ma16175930

**Published:** 2023-08-30

**Authors:** Joanna Liszkowska, Grażyna Gozdecka, Magdalena Sitarz

**Affiliations:** 1 Department of Chemistry and Technology of Polyurethanes, Institute of Materials Engineering, Kazimierz Wielki University, J. K. Chodkiewicza 30, PL 85-064 Bydgoszcz, Poland; 2 Department of Technology and Engineering of the Food Industry, Faculty of Chemical Technology and Engineering, Bydgoszcz University of Technology, PL 85-064 Bydgoszcz, Poland; lipowiec@utp.edu.pl; 3 Syl & Ant Instruments, ul. Pyskowicka 12, PL 44-172 Niewiesze, Poland

**Keywords:** rigid polyurethane–polyisocyanurate PU/PIR foam, instant freeze-dried coffee, color of foams, thermal degradation, biodegradation, climatic chamber, industrial food cocoa

## Abstract

Two series of rigid polyurethane–polyisocyanurate (PU/PIR) foams were obtained. They were modified using powder fillers, such as industrial food cocoa (K5–K15 foam) and instant freeze-dried coffee (KR-KR15) added in amounts of 5, 10 and 15 wt.%. W foam (reference) was obtained without filler. The foams were degraded in a climate chamber for 1 week, 2 weeks or 3 weeks. Appropriate temperature, humidity and UV radiation were set in the chamber, which did not change throughout the degradation process. The foams were also degraded in an oven for two days at 120 °C. The foam tests carried out indicated, among others, on the decrease in compressive strength along with the increase in the residence time of the samples in the chamber. Degraded foams also changed color. Foams containing 5% and 10% of industrial cocoa or freeze-dried coffee were more susceptible to degradation. The addition of 15% coffee or cocoa slows down the degradation process. In the present study, industrial food cocoa and instant freeze-dried coffee were used as modifiers of rigid PU/PIR foam. These fillers have two functions: they accelerate the biodegradation of foams and have antioxidant properties.

## 1. Introduction

Sources [1] state that in 2015 the world value of polymer foams was over 100 billion and sales were 22 million tons. Consumption in 2019 was expected to reach 25.3 million tons. However, it has been estimated [2] that in 2020 their market value will increase to USD 38 billion. It is projected to be worth USD 54 billion in 2025.

Foam is a lightweight material. Therefore, it is widely used, replacing other, heavier materials, e.g., in the packaging industry, aviation, transport, construction, military applications, airplanes, furniture, in insulation, toys and the automotive industry [3,4,5,6,7,8,9,10,11,12,13,14]. They are used for products that come into contact with food. They are used in footwear, furniture and other industries.

Trends in sustainable development point toward the production of ecological and environmentally friendly materials [15,16]. The high cost of polyurethane foam production requires the manufacturer to look for renewable raw materials or to use natural raw materials that are often byproducts or waste from various industries [17]. Growing concerns about the environment and limitations of fossil resources also oblige the production of renewable PU foams [18,19,20].

In order to modify and obtain PU with the desired properties, various types of fillers of plant origin are used [21,22]. Recently, it has become popular to add pulp to PU [8], which in combination with polyurethane (PU) provides a good insulating composite. Biofoams created using agricultural waste were studied by Zhang et al. [23]. They showed that the obtained biofoams with rapeseed, wheat and corn straw displayed excellent thermal stability with heating until 240 °C, stability in water, degradability and elastic ability. In the production of biopolyol foams, wheat straw, oilseed rape straw, rice straw and corn stover particles were used as reinforcement [24]. These materials can be used in isolation.

In order to shorten the life of PU plastic, biodegradation or accelerated aging are used. A study on the thermal conductivity of foams aged at 20–22 °C, avoiding direct sunlight, was conducted by Yakushin [25]. A 19% and 20% increase in thermal conductivity of pouring and spray foams was observed after 180 days of getting older. Biopolyols are also used to increase the degradability of foam [21,26]. The polyurethane foam obtained by Szpiłyk [27] was 70–80% biodegraded in soil within 28 days. The polyol used for its production was synthesized from cellulose, glycidol and ethylene carbonate in water.

Thermal conductivity also changes during aging. The type of these changes is related to the type of blowing agent used [28,29,30]. Most of the published data relate to the aging of foams with blowing agents of the second or third generation.

Currently, most developed countries are accelerating the production of bioplastics [31]. They are considered less harmful than traditional materials. Soares et al. [32] dissolved used coffee grounds in PEG 400 and glycerol using sulfuric acid as a catalyst. They have replaced petrochemical polyols in the synthesis of foams.

This research is a continuation of research on the effect of antioxidants in the form of cinnamon, ground coffee, green coffee on the properties of rigid polyurethane–polyisocyanurate (PU/PIR) foams [33,34]. The studies carried out so far have also concerned the impact of instant cocoa, instant coffee and instant cinnamon, used in the cosmetics industry, on the properties of foams [22]. Since these are not widely available fillers, it was decided to replace them with available freeze-dried instant coffee and food cocoa. In the present study, industrial food cocoa and instant coffee were used as modifiers of rigid PU/PIR foam. The aim of this research was to determine the effect of industrial cocoa DecoMorreno (K) and instant coffee Robusta (KR) on the properties of PU/PIR foams. We determined how the abovementioned modifiers affect the process of photodegradation and biodegradation of foams. The influence of K and KR on UV degradation was investigated. The antiaging properties of these fillers in foams were determined. The work is a continuation of research on the effect of plant fillers on the properties of RPU/PIR. So far, the results of research on foams containing ground coffee [35] and cocoa, green coffee and cinnamon extracts, which are antioxidant additives used in the cosmetics industry, have been published [22].

This article describes foams containing edible, industrial food cocoa (K) and soluble freeze-dried coffee (KR), which are widely available on the market (Table 1). This research aimed at determining the influence of K and KR on degradation process of PU/PIR foams that were subjected to degradation in a degradation chamber. In order to test their thermal stability, the foams were also subjected to thermal property testing (TGA thermogravimetric analysis) and drying in a dryer with forced air circulation. Fourier transform infrared (FTIR) spectroscopy was also performed. Changes in the color of the foams under the influence of temperature, moisture and light were also examined. Physicomechanical tests of the foams were performed.

The aim of this research was to use cheaper and widely available raw materials for PU/PIR foam in the form of food cocoa and freeze-dried instant coffee in comparison with their cosmetic concentrates used so far. These raw materials were to replace cosmetic cocoa and coffee concentrates used in cosmetics as antioxidants. The K and KR modifiers used here in the amount of 10 wt.% act as biofillers, i.e., they have a biodegradable effect. With the addition of 15 wt.%, they have an antioxidant effect.

## 2. Materials and Methods

### 2.1. Materials

The following ingredients were used to synthesize the foams:

- Technical polymeric diisocyanate Purocyn B (supplied by Purinova, Bydgoszcz, Poland), whose main component was 4,4′-diphenyl-methane-diisocyanate (MDI). Density of Purocyn B at temperature of 25 °C was 1.23 g/cm^3^, viscosity was 200 mPas, and content of -NCO groups was 31.0%. Polyether and diisocyanate were characterized in accordance with appropriate standards like ASTM D 2849-69 and ASTM D 1638–70.
- Rokopol RF-551—sorbitol oxyalkylation product (hydroxyl number—420 mg KOH/g, molecular weight—650 g/mol, functionality—4.5 (PCC Rokita S.A., Brzeg Dolny, Poland).
- Stabilizer of foam structure—poly(oxyalkilene siloxane) surfactant Tegostab 8460 (Evonik, Essen, Germany).
- Catalytic system: (a) trimerization catalyst—33% solution of anhydrous potassium acetate (Chempur, Poland) in diethylene glycol (Chempur, Poland); (b) polyurethane bond catalyst—33% solution of DABCO (1.4-diazabicyclo[2.2.2]octane (Alfa Aesar, Haverhill, MA, USA) in diethylene glycol (Chempur, Poland).
- Blowing agent—carbon dioxide produced in situ in the reaction between water and isocyanate groups.
- Flame retardant—Roflam P tris(2-chloro-1-methylethyl) phosphate (V) (PCC Rokita S.A., Henryka Sienkiewicza str. 4, 56-120 Brzeg Dolny, Poland).
- Modifiers: (a) instant freeze-dried coffee Green Bellarom (Lidl, Bydgoszcz, Poland); (b) industrial food cocoa DecoMorreno (producent code 000136, Lidl Bydgoszcz, Poland) (Table 1).

Green Ballerom is 100% Robusta, based on beans from Indonesia or Vietnam. Instant coffee contained 45 wt.% of polyphenols. Coffee or cocoa contain phenolic and chlorogenic acids and flavonoids [36]. The rest of the composition was cellulose, lignin, polysaccharides [37,38,39,40,41,42,43].

According to the literature [36], cocoa contains phenols such as flavanols (including epicatechin), procyanidins, and other flavonoids. In addition, cocoa is supposed to contain methylxanthines with an immunomodulatory effect: theobromine and caffeine (about 1.2%) [44]. According to the manufacturer [45], DecoMorreno cocoa does not contain polyphenols. The diameter of cocoa beans is less than 0.1 mm. However, the manufacturer of Robusta coffee shows that it contains such polyphenols as: flavonoids, alkaloids, terpenoids, phenolic [46]. The diameter of coffee is 2–4 mm. The structure and properties of polyphenols have been discussed, among others, in [39].

### 2.2. Synthesis of the Rigid PU/PIR Foams

Foam formulations (Table 2) were calculated based on the reference sources [47,48]. Detailed calculations are provided in articles [49,50]. PU/PIR foams were prepared at Laboratory scale using a one-step method from the two-component system (A and B) in the chemical equivalent ratio (R) of NCO groups to OH groups equal 3.7:1 (isocyanate index—70) [51]. An excess of polyisocyanate (3.7 R instead of 3.0 R) was used for reaction 0.7 R polyisocyanate with water. The NCO group chemical equivalent (R) was calculated according to Equation (1):

(1) $$R_{NCO}=\frac{4200}{31\%NCO}$$

where % NCO—Content of NCO group in polyisocyanate raw material (%).

The chemical equivalent of hydroxyl group (R) was calculated according to the following equation:

(2) $$R_{OH}=\frac{56100}{HN}$$

where HN—hydroxyl number of Rokopol RF-551 (mg KOH/g).

Polyol premix—Component A was obtained by the precise mixing of the appropriate amounts of ingredients listed in point 2.1: polyol (66.80 g), trimerization catalyst (8.00 g), polyurethane bond catalyst (3.20 g), flame retardant (47.60 g), surfactant (5.40 g), distilled water (3.15 g). Component B was polyisocyanate (of 250.60 g). Components A and B were mixed at respective mass ratio using a mechanical stirrer (1800 rpm, 10 s) and poured into an open cuboidal mold with internal dimensions of 190 mm × 190 mm × 230 mm. The reference foam (W_0t) was obtained in this way. W foam was modified by addition of plant fillers (instant coffee—KR, or cocoa—K) in amounts of 5, 10 or 15 wt.% (Table 2). The amount of filler was calculated in relation to the sum of the polyol and polyisocyanate. Six modified foams were obtained: KR5_0t, KR10_0t, KR15_0t, K5_0t, K10_0t, K15_0t.

### 2.3. Methods

The foams were aged for 24 h at room temperature before cutting out the samples for characterization. Then, the samples were cut with an accuracy of 0.1 mm and weighed with an accuracy of 0.01 g.

#### 2.3.1. Analysis of Foaming Process

The foams were foamed according to ASTM D7487 13e1. [48]. The characteristic foaming times were measured using an electronic stopwatch: creaming time, free rise time, gelling time, dryness time [33,35,47,48]. The maximum reaction temperature (T_max_) was also measured using an electronic thermometer (Browin 145709), which was placed inside the obtained PU/PIR foams.

#### 2.3.2. Thermostating in a Dryer

One of the methods of accelerated foam aging was placing cubic samples with a side of 50 mm in a dryer with forced air circulation (48 h, 120 °C) in accordance with ISO 1923:1981 and PN-EN ISO 4590:2016-11. In this way, it was checked whether the samples changed in linear dimension in line with the foam growth (Δl_w_) and opposite to the foam growth (Δl_p_)—Equations (3) and (4). The change in geometric volume (ΔV, Equation (5)) and the mass loss (Δ_m_, Equation (6)) were also calculated.

Δl_w_ = [(l_w_ − l_0_)/l_0_] × 100% (3)

Δl_p_ = [(l_p_ − l_0_)/l_0_] × 100% (4)

where l_0_—length of the sample before thermostating, according to the direction of foam free rise (mm); l_w_—length of the sample after thermostating, according to the direction of foam rise (mm); l_p_—length of the sample after thermostating, against the direction of foam rise (mm).

ΔV = [(V − V_0_)/V_0_] × 100% (5)

where V_0_—geometrical volume of the sample before thermostating (mm^3^); V—geometrical volume of the sample after thermostating (mm^3^).

Δm = [(m − m_0_)/m_0_] × 100% (6)

where m_0_—mass of the sample before thermostating (g); m—mass of the sample after thermostating (g).

#### 2.3.3. Aging in a Climate Chamber

Mechanical properties play an important role in the mentioned applications of foams (construction, furniture, aviation, electronics, transport). They also include resistance to aging processes under the influence of atmospheric factors. A test involving controlled action of destructive factors, elevated temperature, humidity and UV radiation was carried out [49,50,52]. The foam aging test under the influence of the abovementioned factors was measured at fixed time intervals: 7 days—1t series of foams, 14 days—2t series and 21 days—3t series. The samples were placed in a heated climatic chamber (DYCOMETAL CCK, model CCK-40/300 NG, Es-tor L.T.D., Poznan, Poland) (50 °C, relative humidity 70%, UV radiation intensity 320.86 W/m^2^) [22]. The heating was carried out in continuous mode, without opening the chamber. Testing in a climatic chamber resulted in deterioration of the physical and mechanical properties of the tested samples [53,54]. Although it was not a complete reflection of degradation in the natural environment, it allowed for the assessment of, for example, changes in thermal properties (DSC), changes in chemical structure (FTIR), determination of the coefficient of variation of compressive strength (CV). After the specified time of degradation, the samples were removed from the chamber and the degraded area was assessed for selected properties. The obtained results were compared with the results of non-aged foams (0t).

#### 2.3.4. Apparent Density

Five 50 mm cube samples from each series (ISO 845:2006) were used for the test. The apparent density was the ratio of foam mass to its geometric volume.

#### 2.3.5. Compressive Strength

The compressive strength was measured on five cubic samples (from each series) with a side length of 50 ± 1 mm (N-93/C-89071; ISO 844:2014) using the Instron 5544 universal testing machine. The samples were subjected to 10% compressive strength: in line with the direction of growth (CSa) and contrary to the direction of growth (CSb).

The aging resistance in relation to the coefficient of variation of compressive strength (CV) was calculated from Equation (7) [53,54]:

CV = (W_x_/W_0_) × 100% (7)

where W_0_—compressive strength measured before foam degradation (kPa); W_x_—compressive strength measured after foam degradation (kPa): x = 7, 14 or 21 days.

#### 2.3.6. Foam Structure (SEM)

Scanning electron microscope “Phenom XL” (ThermoFisher Scientific, Schiphol, The Netherlands) was used. The source of electrons was CeB6, a SEC sputter pioneer was used, model: MCM-100P Ion Sputter Coater, MSA system software. The samples were dusted with a gold layer (sputtering time 40 s). The studies were performed at 200x magnification. SEM allowed for the cell height (H) and width (W) to be measured. The cell width and height results were used to calculate the anisotropy coefficient (AC) (Equation (8)):

(8) $$AC=\frac{H}{W}$$

The base H and W values were used to calculate the cel/whole surface area (SA)—Equation (9).

(9) $$SA=H\times W({\mathrm{mm}}^{2})$$

#### 2.3.7. Chemical Structure (FTIR)

Infrared spectra obtained using a Nicolet iS10 FTIR spectrometer with a DTGS detector (Thermo Fisher Scientific, Waltham, MA, USA) allowed for the evaluation of the PU/PIR chemical structure. The spectroscopic range was 4000–400 cm^−1^, maximum resolution < 0.4 cm^−1^.

#### 2.3.8. Measurement of Foam Color

Spectrophotometer SF80 (TRI-COLOR sp. z o.o., Jodłowa str. 50, 32-095 Narama, Poland) was used to measurement of change of colors. Three L*, a*, b* values were measured for each sample. The a*, b* and L* values describe a path (a color trajectory) in the CIELAB color space [55]. Equation (10) was used to calculate the difference between two colors in space:

(10) $$∆E=\sqrt{{\left(∆L*\right)}^{2}+{\left(∆a*\right)}^{2}+{\left(∆b*\right)}^{2}}$$

where L*—brightness (vertical axis of the system); a*—amount of red (positive values “a*”), amount of green (negative values “a*”); b*—amount of yellow (positive values) or blue (negative values).

#### 2.3.9. Softening Point

The softening point was performed with the Vicat apparatus in accordance with the DIN 53424 standard. The temperature at which a standardized needle is immersed into the surface of the test sample to a depth of 1 mm was determined; samples were subjected to a compressive load (24.52 kPa) at temperature increasing at a constant speed (50 °C/h). The weighted portion was 80 mg.

#### 2.3.10. Retention

The test of the sample combustion residue (retention) was carried out using a simplified chimney test (Butler’s vertical test) according to ASTM D3014-73. The apparatus used to test flammability according to the vertical test consists of a vertical column with dimensions of 300 × 57 × 54 mm; three of the walls are made of sheet metal and the fourth is a movable glass. The determination was carried out on six samples with dimensions of 150 × 19 × 19 mm. Before burning, the samples were weighed with an accuracy of 0.001 mm and then placed inside the chimney. A flame from a propane–butane burner was applied to the sample for 10 s. The burner was removed, and the samples were reweighed. The combustion residue was calculated according to Equation (11):

(11) $$R=\frac{m}{m_{0}}\times 100\%$$

where

R—combustion residue (retention);

m_0_—mass of the sample before combustion [g];

*m*—mass of the sample after combustion [g].

#### 2.3.11. Water Absorption and Absorbability

Water absorption (Ch) and absorbability (*N*) were determined in accordance with ISO 280 2896:2001. Samples with dimensions of 150 × 150 × 25 mm were used for the tests. The foams were soaked in distilled water for 24 h. Water absorption was calculated from Equation (12) and water absorbability from Equation (13).

(12) $$\mathrm{Ch}=\frac{mWA-mD}{mD}\times 100\%$$

where *mWA*—mass of the sample after surface drying (g).

(13) $$N=\frac{mA-mD}{mD}\times 100\%$$

where *mA*—mass of the sample after immersion in distilled water (g); *mD*—mass of the dry sample (g).

This method is applicable to all rigid porous plastics that do not react with and do not dissolve in water.

#### 2.3.12. Closed Cell Content

The content of closed cells was determined in accordance with the PN-ISO 4590:1994 standard, method II, using foam samples with dimensions of 100 × 30 × 30 mm. This method consists of determining the relative pressure drop, previously calibrated for volume standards, from the difference of indications on the manometer scale, one arm of which is open to the atmosphere. This measurement method is designed to determine the percentage of cells enclosed in rigid porous materials.

#### 2.3.13. Thermal Resistance

TGA (thermogravimetric analysis) tests were carried out using a Q500 thermobalance (TA Instruments, New Castle, DE, USA) in a nitrogen atmosphere (temperature range from 0 to 1000 °C, temperature change rate 10 °C/min). The weight of the sample was about 21 mg. Based on the TG curves, the temperature of 5% (T5), 20% (T20) and 50% (T50) loss of the initial mass of the sample was determined. From the differential DTG curve (first derivative of the TG curve) the T_max_ values (the temperature of the fastest mass loss) were also determined.

#### 2.3.14. Standard Deviation from the Arithmetic Mean

For individual values, the standard deviation from the arithmetic mean (*σ*) was calculated according to Equation (14).

(14) $$\sigma =\frac{\sqrt{{\left(x_{1}-X\right)}^{2}+{\left(x_{2}-X\right)}^{2}+\ldots +{\left(x_{n}-X\right)}^{2}}}{n}$$

where *x*_1_, *x*_2_,…*x_n_*—property value data

*X*—the arithmetic mean of the *x*_1_,…*x_n_* values

## 3. Results and Discussion

### 3.1. Foaming Process and Density

Research on rigid foams is related to the requirements of recipients of foams with different physical, chemical and structure properties. It is important to know the PU density, flammability, water absorption, thermal conductivity, brittleness, compressive strength, etc. The research results confirm that there is a close relationship between the properties of the obtained modified foams and their physical and chemical structure as well as the type and amount of vegetable filler used.

During the preparation of RPU/PIR, the times of subsequent stages of the foam formation process were determined. The processing parameters of the foams increased with the increasing content of the filler, except that for foams modified with soluble coffee (KR) this increase is slightly greater (e.g., free rise time from 30 s to 55 s) compared to foams filled with food cocoa (K) (from 30 s to 43 s)—Table 3. When foaming the foam composition with a specific concentration of the trimerization catalyst and urethane bond catalyst, the creaming time of all the compositions was 10 s. The increase in string gelation time was affected by the addition of cocoa or coffee. String gel time increased with the increasing content of instant coffee or cocoa from 23 s, respectively (reference foam, W_0t), up to 37 s (KR15_0t, foam with 15% instant coffee) and up to 29 s (K15_0t, foam with 15% cocoa).

Determining individual times makes it possible to evaluate the course of the process, and possibly modify it [55].

The maximum reaction temperature (T_max_ ) reached its highest value for the 5% content of plant fillers in both cases (K5_0t and K5_0t foam, 160 °C and 153 °C, respectively). With higher contents of K and KR fillers, T_max_ decreases.

Apparent density is a very important factor in the usability of RPUFs [10]. The apparent density of the obtained foams containing coffee (KR) is about 37–41 Kg/m^3^ and does not change significantly in relation to the density of the foam W. On the other hand, the apparent density of foams with cocoa (K) slightly decreases compared to the density of the foam W and varies in the range of about 36–30 Kg/m^3^. Foams of such density can be used to pack heavier and less delicate products, such as metal components, pharmaceutical or electronic products. They provide better protection against shocks and impacts, which is important for ensuring the quality and durability of products [10,56,57].

### 3.2. SEM

In order to determine the influence of cocoa and coffee on the size and anisotropy of foam cells, an SEM study was performed, showing the incorporation of fillers into the foam structure.

**Figure 1:**
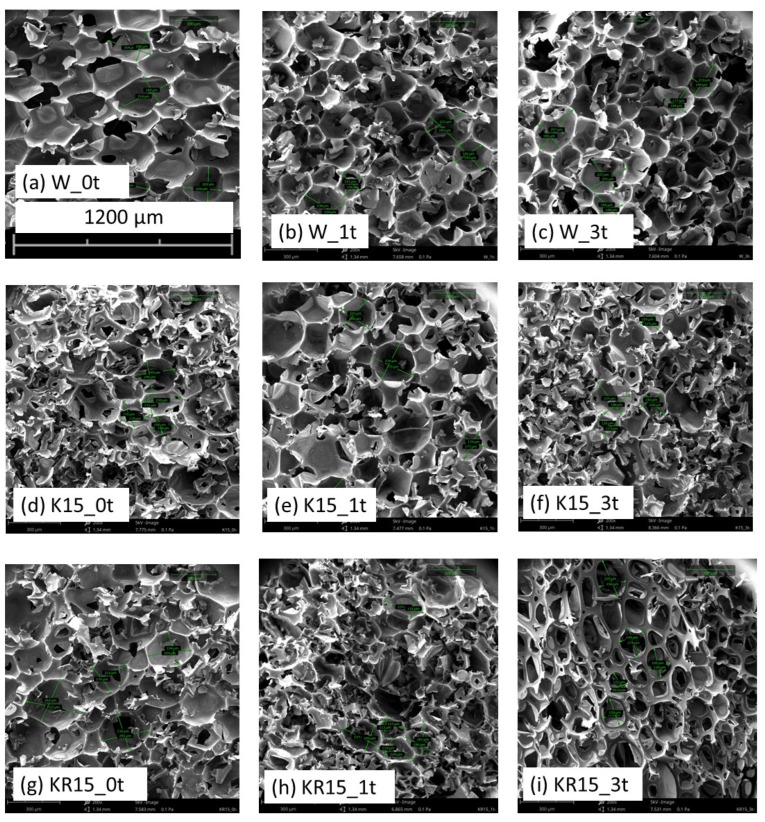
SEM of W, K and KR foams: magnification 200×, 5 kV.

Nondegraded foams (0t) and degraded foams 1t and 3t modified with the highest content of fillers—cocoa (K15) and instant coffee (KZ15)—were subjected to the SEM study—Figure 1. Foams filled with 15% coffee or cocoa (KR15 and K15) are characterized by cells with a smaller diameter [25]. The probable cause of the decrease in cell diameter was the incorporation of filler particles (cocoa and coffee) into the foam pore walls. In addition, when foaming the composition, there was sufficient time for the formation of larger cells for the W foam and insufficient time for the modified foams (KR15 and K15) Table 4. Due to the increasing viscosity and density of the polyol masterbatches (organoleptic assessment), a longer mixing time of components A and B is probably necessary with the increase in the amount of fillers.

During the degradation of the W foam, the average area of cells decreases from 0.047 mm^2^ (W_0t) to 0.037 mm^2^ (W_3t)—Table 4. The probable cause was the incorporation of coffee and cocoa particles into the foam pore walls with their simultaneous opening, which was confirmed using microscopic images (Figure 1). The opening of the pores at the same time resulted in an increase in the absorbency and absorbability of the foams. 

The results of SEM micrograph analyses show that the anisotropy coefficient (AC) takes values close to one. This means that the shape of the foam cells is almost spherical [57]. It was observed that the diameter of the foams (SA) decreases with the increase in the degradation time of the foams. This is probably due to the incorporation of coffee or cocoa particles into the foam structure and cracking of the foam walls.

### 3.3. Aging Measurement

Changing a linear dimension according to the direction of foam growth (Δl_z_) did not exceed the value ± 1.4%; the change in linear dimensions against the direction of foam growth (Δl_p_) was from +0.1 to +2.94%; the change in geometrical volume (ΔV) fluctuated between −0.34 and −4.68—Table 5. None of the values Δl, ΔV exceeded 5%, which is required in construction. Mass loss (Δm) was between +0.6 and +5.63.

### 3.4. Retention, Fragility, OI

The addition of cocoa and coffee particles and their incorporation into the foam structure had no significant effect on the residue after foam combustion (R)—Table 6. A slight increase in R was observed in KR foams (84.68%, KR10) and a slight decrease in R in K foams (78.17%, K15) in relation to the R value was observed in the W foam (83.44%).

A slight decrease in the brittleness of the K15 (14.09%) and KR15 (13.79%) foams was observed in relation to the brittleness value of the reference foam W (16.42%). This was due to the incorporation of cocoa and coffee into the foam structure.

Oxygen index (OI) ranges from 24.7% (W foam) to 24.2% (KR15 foam) and up to 23% (K15 foam). It can be seen that the structure of the foam (Figure 1) contributed to the decrease in OI for the cocoa-containing foam.

### 3.5. Conductivity versus Foam Density and Cell Size

The basic indicator of thermal insulation properties of foams is thermal conductivity [57]. Thermal conductivity depends on cell orientation and size, thermal conductivity of blowing agent in the cells, density and closed cells content and thermal conductivity of modifiers.

Theoretical and practical works have shown that the impact of the radiative constituent on the overall thermal conductivity strongly depends on cell sizes. The reduction in cell sizes yields smaller radiative contributions in thermal conductivity [25,58,59,60,61,62].

The thermal conductivity coefficient of the reference foam was 0.0289 W/mK, while for the other foams its increase to 0.035 W/mK was observed. Such a coefficient allows for the use of the obtained materials as protection for products against extreme temperatures during transport.

### 3.6. Water Absorption, Absorbability, Closed Cell Content

The hydrophobicity of PU is influenced by the chemical and physical structure. High cross-linking density reduces the mobility of the PU molecule and hinders water penetration [63].

Comparing the obtained results with the tests carried out so far, it can be suggested that the cells of foams containing cocoa or coffee (Figure 1, Table 4) did not decrease at all, but simply cracked the cell walls, which proves the growth of open cells (Table 7). Thus, the results of the average cell diameter of the foams (Table 4) can be considered as a measure of the cell openness and the increase in the number of holes on a given surface of the foam. Thus, with the increase in the content of open cells (Z) (Table 7), the absorbency (Ch) and water absorption (N) of the foams increase, and the compressive strength decreases (Table 8).

The softening point value ranges from 171 to 192 °C, the highest is for the K5_0t sample and decreases with the increase in cocoa in the foam and increases with the increase in the content of freeze-dried coffee in the sample (for KR15_0t and W_0t foam it is 184 °C)—Table 7.

Water absorption (Ch, Table 7) did not exceed 5% (except for the W_0t and KR15_0t samples), which is why the foams are useful in industry. The content of closed cells decreased from 87% to 9.6% (KR15_0t) and to 9% (K15_0t).

### 3.7. Compressive Strength

The addition of K and KR antioxidants reduces the compressive strength both contrary to the direction of growth (CSa) and in line with the direction of growth (CSb)—Table 8. Also, extending the residence time of samples in the degrading chamber (from 0t to 3t) causes a decrease in CSb of individual samples, e.g., for W foam from 251.63 kPas (W_0t) to 140.60 kPas (W_3t); for K5 series foams from 167.82 kPas (K5_0t) to 90.95 kPas (K5_3t)

The compressive strength measured in line with the direction of growth (CSb) of PU materials used for wall insulation is usually approx. 0.15 MPa [64].

Knowing the results of the aging tests, we were able to determine the coefficient of variation (CV) in relation to aging [50,52]. For our tests, the CV of the compressive strength was determined—Table 8. CV1 was calculated as a ratio of compressive strength of 0t and 2t series foams and CV2 as a ratio of compressive strength of 0t and 3t series foams. The choice of this property for the assessment of aging was made due to its importance in the application of polyurethane foams in civil engineering. The CV1 and CV2 of the tested foams was 60.3 and 55.9 for the W-series foams, respectively. The addition of coffee or cocoa increased CV1 to 76.6 and 78.8 (KR15_0t and K15_0t) and increased CV2 to 69.6 for cocoa foam (K15_0t) and decreased CV2 to 51.3 for coffee foam (KR15_0t).

### 3.8. FTIR

#### 3.8.1. Influence of Degradation Time on Absorbance of W, K5 and KR5

**Figure 2:**
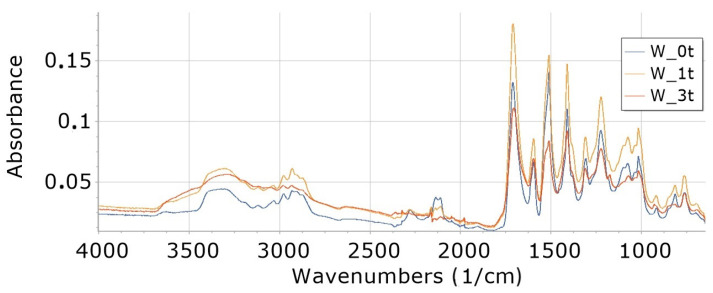
FTIR showing the course of degradation of W foams: nondegraded (W_0t), degraded 1 week (W_1t) and 3 weeks (W_3t).

**Figure 3:**
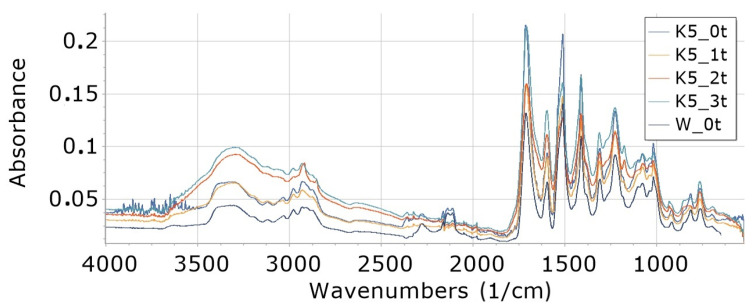
FTIR for foam content 5% food cocoa, undegraded (K5_0t) and degraded one week (K5_2t), two weeks (K5_2t) and three weeks (K5_3t).

**Figure 4:**
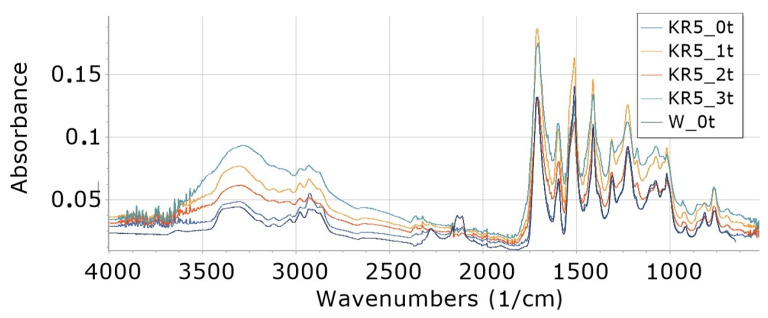
FTIR for foam content 5% freeze-dried coffee, undegraded (KR5_0t) and degraded one week (KR5_1t), two weeks (KR5_2t) and three weeks (KR5_3t), and reference foam W_0t.

**Figure 5:**
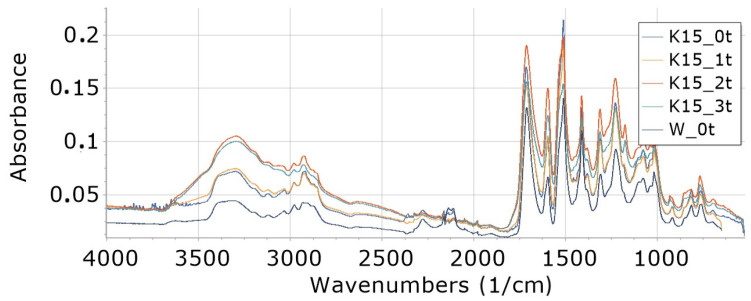
FTIR for foam content 15% food cocoa, undegraded (K15_0t) and degraded one week (K15_1t), two weeks (K15_2t) and three weeks (K15_3t).

**Figure 6:**
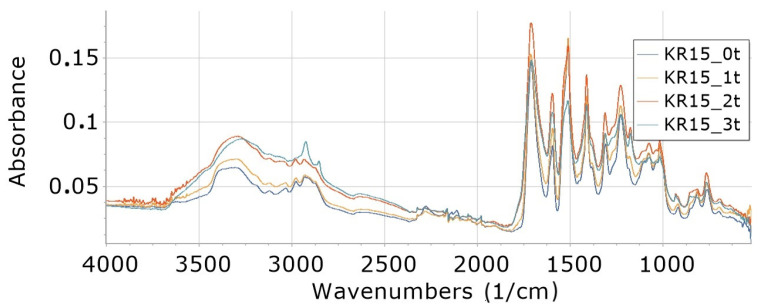
FTIR for foam content 15% freeze-dried coffee, undegraded (KR15_0t) and degraded one week (KR15_2t), two weeks (KR15_2t) and three weeks (KR15_3t), and reference foam W_0t.

**Figure 7:**
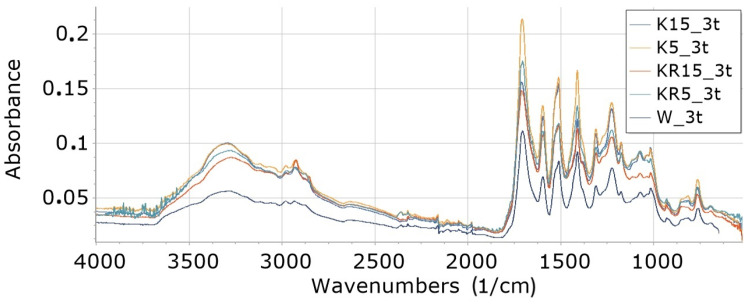
FTIR for foams degraded three weeks (3t): reference foam (W), foam content 5% and 15% food cocoa (K5, K15) or freeze-dried coffee (KR5, KR15).

Interpretation of FTIR spectra was made on the basis of [65,66]. FTIR analysis of foams (Figure 2, Figure 3, Figure 4, Figure 5, Figure 6 and Figure 7) showed the presence of bonds, e.g., such as N–H (3325 cm^−1^, 1596 cm^−1^, 1512 cm^−1^), C=O, –N=C=O (2276 cm^−1^), CH (2930 cm^−1^), –N=C=N (2137 cm^−1^) in a urethane bond (1713 cm^−1^) and isocyanurate ring (1411 cm^−1^), C–O (1076 cm^−1^), and –C=N in the trimer (1225 cm^−1^). These are bonds that occur in the structure of polyurethane–polyisocyanurate foam.

The figures (Figure 2, Figure 3, Figure 4, Figure 5, Figure 6 and Figure 7) show the change in the absorbance of W, K and KR foams depending on the degradation time (0t, 2t, 3t) or the amount of modifier (0%, 5%, 15%).

In the range of wavenumbers 4000–2400 cm^−1^ for the reference foam (W), an increase in the absorbance of individual bonds of the degraded foam was observed for 1 week (W_1t) relative to the absorbance of nondegraded foam (W_0t)—Figure 2. However, a longer degradation time (3 weeks) causes a decrease in the absorbance of the W_3t foam in relation to the W_2t foam in this range of wavenumbers. For wavenumbers 2400–800 cm^−1^, a decrease in the absorbance of the degraded foam was observed for 3 weeks (W_3t) below the value of the absorbance of bonds of the foam W_0t (not degraded).

In the case of a small (5%) cocoa content in the foams (K5), an increase in the absorbance of individual bonds was observed with an increase in the degradation time from 0t to 3t in the wavenumber range of 4000–2000 cm^−1^—Figure 3. In the wavenumber range from 2000 cm^−1^ up to 800 cm^−1^, the largest increase in absorbance is observed for foams degraded for 1 week (1t), and a further increase in degradation time (2t and 3t) causes a decrease in absorbance in this range of wavenumbers.

In the case of low (5%) content of freeze-dried coffee in foams (KR5), an increase in the absorbance of individual bonds was observed with an increase in degradation time from 0t to 3t in the wavenumbers ranging 4000–2000 cm^−1^—Figure 4. In the range of wavenumbers from 2000 cm^−1^ to 800 cm^−1^, the largest increase in absorbance was observed for foams degraded for 1 week (1t), and a further increase in degradation time (2t and 3t) caused a decrease in absorbance in this range of wavenumbers.

#### 3.8.2. Influence of Degradation Time on Absorbance of K15 and KR15

From the graph (Figure 5) it can be seen that with the increase in the degradation time (from 0t to 2t) of foams containing more, i.e., 15% cocoa (K15_0t to K15_2t), the absorbance of individual bonds increases. On the other hand, further degradation of 3 weeks (K15_3t) causes a decrease in absorbance again.

From the graph (Figure 6), it can be read that with the increase in the degradation time (from 0t to 2t) of foams containing 15% freeze-dried coffee, the absorbance of individual bonds in relation to the foam increases. On the other hand, further degradation of 3t causes a decrease in absorbance again.

#### 3.8.3. Influence of Type and Amount of Filler on Absorbance of Foam Degraded 3 Weeks (Foams 3t)

The addition of 5% cocoa causes accelerated degradation of the K5_3t foam in relation to the rate of degradation of the W_3t foam, because the absorbance of individual bonds of the K5_3t foam increases compared to the absorbance of these bonds in the foam without cocoa (W_3t). However, the addition of 15% cocoa (K15_3t foam) caused a decrease in absorbance compared to the foam containing only 5% of degraded cocoa (K5_3t foam)—Figure 7. The content of 15% cocoa slows down the degradation of the foam compared to 5% cocoa addition. The addition of 15% freeze-dried coffee worked similarly. An increase in the absorption of KR5_3t foam bonds was observed in relation to W_3t, but a decrease in relation to KR15_3t. In the case of 3-week degradation, both cocoa (K) and freeze-dried coffee (KR) slowed down the decomposition of bonds in foams. This may indicate that these additives have antioxidant properties in PU/PIR foams.

### 3.9. Color of Foams

From the test results (Table 9), it can be read that the transparency of the foams increases with the increase of foam degradation time. Foams degraded for 3 weeks (marked as 3t) are characterized by the highest transparency (L) in the range of about 43–54, while the nondegraded foams (marked as 0t) are characterized by the highest transparency (marked as 0t) in the range of about 71–83. Foams degraded for 2 and 3 weeks have the highest the value of *a* (approximately 16–23), which represents the color red. Nondegraded foams have a value close to zero, but in the case of the content of 15% cocoa (K15) or 15% coffee (KR15), there is a slight decrease in the value of *a* in relation to the value of foams containing 5% of these fillers (K5 and KR5). The largest increase in red color was observed for W foam from -0.59 (W_0t) to 16.97 (W_3t).

The content of yellow color (b) of degraded foams (1t, 2t and 3t foams) significantly increases in relative to the *b* value of nondegraded foams (0t foams). The values *b* increase, for example, for K foams from 15.85 (KR5_0t) to 47.65 (KR5_3t). This means that the addition of fillers (K and KR) accelerates the degradation of foams more than twofold. However, as in the case of the value of *a*, a decrease in the value of *b* is observed with an increase in the content of K and KR in the foams. That is, the increased amount of cocoa and coffee inhibits the degradation of K15 and KR15 foams in relation to K5 and KR5.

Extending the degradation time causes an increase in the amount of red and yellow color, i.e., the decomposition of the foams increases. An increase in these colors (values *a* and *b*) indicates an increased degradation of the foams with increasing degradation time. The decrease in the value of *a* and *b* with the increase in the amount of coffee (KR) or cocoa (K) with the longest degradation time (3t—3 weeks) means that the increased amount of these fillers in the foams inhibits the decomposition of the foams and slows down the degradation.

The ΔE values decrease by about 20% with increasing foam degradation time. The highest value was observed for W_0t equal to about 85 and the lowest for KR15_3t equal to about 64. This study confirmed that the content of freeze-dried coffee food cocoa increases the resistance of foams to degradation (Table 9, Figure 7).

### 3.10. Thermal Properties

In the case of degradable foams, understanding their interaction with the environment is extremely important, because they can end up as household waste that is exposed to the environment [67]. Stability and degradation are among the most important properties when it comes to the application of degradable foams [67,68,69].

Thermal stability is enhanced by crosslinking, while breaking the main chain affects the thermal degradation of PU [70]. The literature reports that two thermal characterization steps occurred between approximately 50–250 °C due to dehydration of sorbitol [71] and elimination of water [72,73] and 250–350 °C due to degradation of starch chains [71,72,73]. The effect of acid concentration on Tonset and Td was demonstrated (increase in acid concentration decreases them). However, the residual weight increased. Studies have shown similar relationships for glycerol/starch-based foams [74,75]. This was due to the reaction of the -OH group of starch and glycerol with -COOH of citric acid by esterification followed by the formation of substitution ester bonds [73,74,75]. However, foams filled with citric acid have been found to lose their weight in the range of 250–270 °C. This observation was explained as hydrolysis and degradation of the starch due to the further step of hardening under the influence of high temperature [73,76].

**Figure 8:**
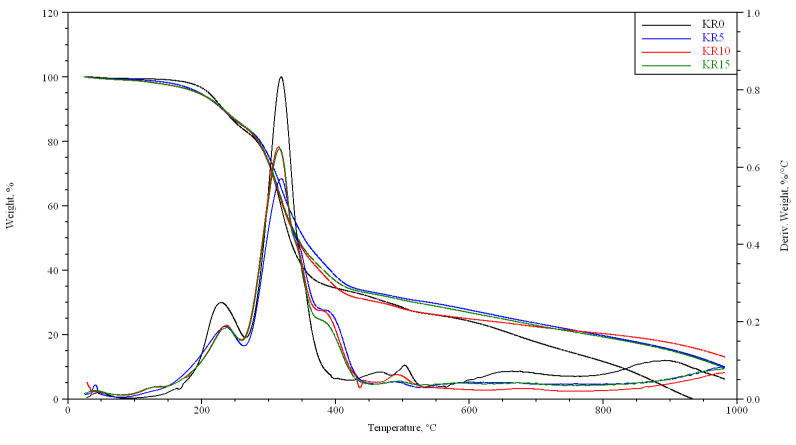
TG and DTA of foams of the KR_0t series.

Thermal properties of foams in nitrogen atmosphere were tested. The results were read from the graphs of the TG and DTA curves (for example, Figure 8 for KR_0t series of foam with coffee) and placed in Table 10. The graphs read, among others: T_1_—start of the change in mass in stage 1; T_2_—beginning of decomposition in stage 2; T5, T20, T50—the temperature at which 5%, 20% and 50% loss of foam mass occurred, respectively; and T_max_—the highest rate of mass loss.

The maximum decomposition temperature of stage 1 (T_1_) of individual samples increases with the time of foam degradation in the degrading chamber, e.g., for KR5 foam containing 5% coffee, it increases from 33.0 °C (KR5_0t) to 57.0 °C (KR5_3t); for K5 foam, it increases from 42 °C (K5_0t) to 46.6 °C (K5_3t). The maximum decomposition temperature of stage 2 (T_2_) of individual samples also increases with the time of foam degradation in the degrading chamber, e.g., for KR5 foam, from 233.0 °C (KR5_0t) to 250.5 °C (KR5_3t); for K5 foam, from 224.7 °C (K5_0t) to 234.3 °C (K5_3t). The maximum decomposition temperature (T_max_) ranges from 308 to 318 °C for all foams.

T5 decreases slightly for each series of foams, e.g., for the K5 series, from 197.1 °C (K5_0t) to 184.6 °C (K5_3t). Similarly, T20 also decreases for individual series of foams. On the other hand, T50 increases for individual foam series, e.g., for the KR5 series, from 353.0 °C (KR5_0t) to 470.9 °C (KR5_3t).

## 4. Conclusions

The content of food cocoa (K) and freeze-dried coffee (KR) increases the resistance of foams to decomposition, which is confirmed by the decrease in the content of red (a*) and yellow (b*) in foams with 15% content of K and KR (K15 and KR15) in relation to the values a* and b* for foams with 5% content of modifiers (K5 and KR5 foams). Thus, a decrease in the group absorbance of the K15 and KR15 foams is observed in relation to the group absorbance of the K5 and KR5 foams.

This study confirmed that the content of freeze-dried coffee and food cocoa increases the resistance of foams to degradation to the content of 15% by mass of these fillers.

The obtained foam material with a filler at an amount of up to 10% can be used as, for example, spacers to protect products against mechanical damage in the packaging industry where discarded foams should degrade as quickly as possible. If the amount of cocoa or coffee is increased to 15%, the stability of the foam increases. It can then be used where the life of such foam should be longer and not subject to rapid decomposition (e.g., during longer transport in nonstandard conditions).

The degradation of the foams in a climatic chamber reduces their compressive strength, which is not always good in rigid foam applications. The obtained foam materials show good thermal insulation properties, which allows for the products to be protected against extreme temperatures during transport.

The conducted research indicates that the addition of a modifier in the form of industrial food cocoa to the foams provides specific results in terms of foam biodegradability and specific results in terms of antioxidant oxidation of these foams. The cosmetic cocoa concentrate used so far can be replaced with cheaper food cocoa. Similar results were observed for the addition of freeze-dried soluble coffee in comparison with the cosmetic concentrate of this coffee, which is not so widely available, and companies reserve its composition.

## Figures and Tables

**Table 1:** Extracts composition: KR—green coffee, K—industrial food cocoa.

Compound	Content In KR Foam	Content in K Foam
Polyphenols (e.g., flavonoids, phenol acids, chlorogenic acid)	45.0%	0.0%
Minerals	4.4%	<0.3 ppm
Carrier to active substances (e.g., lignin, cellulose, polysaccharides) Fat Carbohydrates	About 50.6% - 41%	About 94.9% 10.5% 13.0%

**Table 2:** The content of modifiers in the PU/PIR foams.

	Filler (wt.%)	
Foam	Instant Coffee	Cocoa
W_0t	0	0
KR5_0t	5	0
KR10_0t	10	0
KR15_0t	15	0
K5_0t	0	5
K10_0t	0	10
K15_0t	0	15

**Table 3:** Processing times, maximum reaction temperatures (T_max_) and apparent density of PU/PIR foams.

Foam	Cream Time (s)	String Gel Time (s)	Tack Free Time (s)	Free Rise Time (s)	T_max_ (°C)	Apparent Density (Kg/m^3^)
W_0t	10	21	23	40	142	38.44 ± 3.6
KR5_0t	10	27	30	41	160	37.67 ± 3.6
KR10_0t	10	33	35	45	140	39.36 ± 3.6
KR15_0t	10	37	40	55	149	41.46 ± 3.6
K5_0t	10	27	29	40	153	35.12 ± 3.6
K10_0t	10	28	31	43	146	36.52 ± 3.6
K15_0t	10	29	35	48	139	30.09 ± 3.6

**Table 4:** Results of SEM micrograph analyses.

Foam	H (μm)	W (μm)	AC (−)	SA (mm^2^)
W_0t	231 ± 29	203 ± 30	1.14 ± 0.27	0.047 ± 0.01
W_1t	208 ± 29	193 ± 30	1.08 ± 0.27	0.040 ± 0.01
W_3t	217 ± 29	170 ± 30	1.30 ± 0.27	0.037 ± 0.01
K15_0t	200 ± 29	149 ± 30	1.34 ± 0.27	0.030 ± 0.01
K15_1t	217 ± 29	188 ± 30	1.15 ± 0.27	0.041 ± 0.01
K15_3t	164 ± 29	138 ± 30	1.19 ± 0.27	0.023 ± 0.01
KR15_0t	268 ± 29	215 ± 30	1.24 ± 0.27	0.058 ± 0.01
KR15_1t	205 ± 29	138 ± 30	1.49 ± 0.27	0.028 ± 0.01
KR15_3t	168 ± 29	131 ± 30	1.28 ± 0.27	0.022 ± 0.01

**Table 5:** Results of foam aging measurement * (120 °C; 48 h/2 days; foams K, KR and W).

Foam	∆l_z_ (%)	∆l_p_ (%)	∆V (%)	∆m (%)
W_48	+0.39 ± 0.01	+0.39 ± 0.01	−1.77 ± 0.01	+3.27 ± 0.01
K5_48	+1.38 ± 0.01	+0.98 ± 0.01	−2.93 ± 0.01	+4.27 ± 0.01
K10_48	−1.39 ± 0.01	+1.12 ± 0.01	−3.08 ± 0.01	+4.56 ± 0.01
K15_48	+1.40 ± 0.01	+2.94 ± 0.01	−4.68 ± 0.01	+5.63 ± 0.01
KR5_48	+0.16 ± 0.01	+0.10 ± 0.01	−0.44 ± 0.01	+0.60 ± 0.01
KR10_48	+0.16 ± 0.01	+0.12 ± 0.01	−0.34 ± 0.01	+1.10 ± 0.01
KR15_48	+0.16 ± 0.01	+0.14 ± 0.01	−0.42 ± 0.01	+0.71 ± 0.01

* Description of abbreviations in the text.

**Table 6:** Fragility (K), thermal conductivity (Λ), oxygen index (OI), retention of residue after burning (R) of foams 0t (nondegraded).

Foam	K (%)	Λ (kW/mK) ±0.00006	OI (%vol. of O_2_)	R (%)
W_0t	16.42 ± 6.25	0.0289	24.7 ± 0.52	83.44 ± 2
KR5_0t	18.96 ± 6.25	0.0350	24.4 ± 0.52	84.96 ± 2
KR10_0t	13.97 ± 6.25	0.0350	24.3 ± 0.52	84.68 ± 2
KR15_0t	13.79 ± 6.25	0.0350	24.2 ± 0.52	82.62 ± 2
K5_0t	17.82 ± 1.85	0.0350	24.2 ± 0.52	84.54 ± 2
K10_0t	13.14 ± 1.85	0.0350	23.8 ± 0.52	81.71 ± 2
K15_0t	14.09 ± 1.85	0.0350	23.0 ± 0.52	78.17 ± 2

**Table 7:** Absorptivity (N), water absorption (Ch), closed cell content (Z), softening point (Sp).

Foam	N (%)	Ch (%)	Z (%)	Sp (°C)
W_0t	12.33 ± 13	5.15 ± 4.8	87.0 ± 24	184 ± 6
KR5_0t	23.51 ± 13	2.72 ± 4.8	44.1 ± 24	178 ± 6
KR10_0t	29.98 ± 13	2.86 ± 4.8	32.7 ± 24	180 ± 6
KR15_0t	53.37 ± 13	16.16 ± 4.8	9.6 ± 24	184 ± 6
K5_0t	19.45 ± 13	0.84 ± 4.8	41.1 ± 24	192 ± 6
K10_0t	24.55 ± 13	2.14 ± 4.8	30.2 ± 24	183 ± 6
K15_0t	43.81 ± 13	4.62 ± 4.8	9.0 ± 24	171 ± 6

**Table 8:** Compressive strength: measured contrary to the direction of growth (CSa), measured in line with the direction of growth (CSb); after 0 degradation time (CS0_0t), after 2 weeks (CS_2t) and after 3 weeks (CS3_3t) of degradation; CV1 and CV2—compressive strength ratio.

Foam	CSa_0t (kPas)	CSb_0t (kPas)	CSb_2t (kPas)	CSb_3t (kPas)	CV1 (−)	CV2 (−)
W_0t	128.21 ± 19	251.63 ± 42	151.80 ± 18	140.60 ± 21	60.3 ± 9.6	55.9 ± 9
KR5_0t	101.87 ± 19	167.82 ± 42	134.00 ± 18	90.95 ± 21	79.8 ± 9.6	76.9 ± 9
KR10_0t	129.79 ± 19	199.14 ± 42	99.98 ± 18	89.86 ± 21	50.2 ± 9.6	54.2 ± 9
KR15_0t	83.96 ± 19	131.40 ± 42	88.19 ± 18	67.45 ± 21	67.6 ± 9.6	51.3 ± 9
K5_0t	111.07 ± 19	188.12 ± 42	124.20 ± 18	115.71 ± 21	66.0 ± 9.6	61.5 ± 9
K10_0t	94.45 ± 19	135.81 ± 42	108.55 ± 18	97.14 ± 21	66.0 ± 9.6	71.5 ± 9
K15_0t	76.15 ± 19	126.69 ± 42	99.81 ± 18	88.19 ± 21	78.8 ± 9.6	69.6 ± 9

**Table 9:** Results of measurements color of foams.

Sample	L* (−)	a* (−)	b* (−)	ΔE (−)
Foam 0t				
W_0t	82.07	−0.59	23.74	85.43665
K5_0t	78.96	2.46	17.32	80.87469
K15_0t	71.89	5.4	16.47	73.94994
KR5_0t	83.29	−0.93	15.85	84.78981
Foam 1t				
W_1t	73.01	4.82	37.64	82.28282
K5_1t	63.65	15.17	38.75	76.04613
K15_1t	62.41	16.72	37.93	74.92164
KR5_1t	61.72	17.94	43.81	77.78508
KR10_1t	61.49	14.75	40.93	75.32495
KR15_1t	60.35	11.83	37.02	71.78128
Foam 2t				
W_2t	62.73	4.94	31.58	70.40421
K5_2t	53.53	20.90	44.57	72.72383
K15_2t	51.03	21.22	42.81	69.90741
KR5_2t	51.53	21.69	43.99	71.14012
KR15_2t	45.64	21.82	41.70	65.55923
Foam 3t				
W_3t	53.93	16.97	38.54	68.42337
K5_3t	47.32	22.77	41.71	67.06250
K15_3t	49.02	21.74	40.42	67.15180
KR5_3t	49.10	23.49	47.65	72.34026
KR10_3t	50.74	21.55	42.71	69.73589
KR15_3t	43.45	22.35	41.55	64.13913

**Table 10:** Thermal resistance results: T_1_—start of the change in mass in stage 1; T_2_—beginning of decomposition in stage 2; T_max_—the highest rate of mass loss of PUR-PIR foams; T5, T20, T50—temperature 5%, 20% and 50% loss of mass in relation to the initial mass of the sample; V_1_, V_2_—degradation rate of steps 1 and 2.

Foam	Stage 1		Stage 2						
	Start of the Change of Mass		Start of the Change of Mass						
	T_1_ (°C) ±0.1	Weight Loss (%) ±0.1	T_2_ (°C) ±0.4	Weight Loss (%) ±0.1	T_max_ (°C) ±0.1	T5 (°C) ±0.4	T20 (°C) ±0.4	T50 (°C) ±0.4	Residue at 900 °C (%) ± 0.1
W_0t	45.0	1.0	227.0	30.0	317.0	207.0	292.0	340.0	2.0
W_1t	50.0	0.0	25.02	15.0	318.7	210.9	285.0	331.5	10.0
W_2t	-	-	-	-	-	-	-	-	-
W_3t	-	-	-	-	-	-	-	-	-
KR5_0t	33.0	5.0	233.0	73.0	319.0	198.0	319.0	353.0	5.5
KR5_1t	38.8	34.0	228.2	89.0	309.9	188.8	287.4	371.1	2.7
KR5_2t	46.0	36.0	247.5	72.0	318.0	185.0	276.0	465.7	1.7
KR5_3t	57.0	9.9	250.5	76.0	308.0	186.8	278.6	470.9	18.0
KR15_0t	27.0	5.0	230.0	26.0	317.0	96.0	233.0	344.0	5.5
KR15_1t	53.5	8.0	231.3	42.0	314.5	193.4	276.1	346.6	8.8
KR15_2t	46.2	7.6	243.7	52.3	314.8	188.6	272.8	399.6	13.5
KR15_3t	-	-	-	-	-	-	-	-	-
K5_0t	42.0	33.0	224.7	58.1	312.3	197.1	282.4	365.5	0.8
K5_1t	38.0	5.0	225.1	35.9	365.3	168.2	268.7	365.3	2.2
K5_2t	50.4	12.1	270.0	63.0	316.0	182.6	271.6	387.9	19.4
K5_3t	46.6	10.0	234.3	60.0	314.1	184.6	268.8	414.7	14.7
K15_0t	51.0	2.5	215.6	31.6	311.9	187.7	274.9	327.1	13.0
K15_1t	-	-	-	-	-	-	-	-	-
K15_2t	49.0	36.5	229.1	37.8	308.8	173.3	263.3	369.3	2.7
K15_3t	53.9	8.3	231.4	38.4	308.5	75.3	68.2	367.5	15.4

## Data Availability

The final data will be made available upon contact with the authors.
